# A Systematic Review and Meta-Analysis of High-Frequency Prescription of Zhigancao Decoction Combined with Conventional Western Medicine in the Treatment of Chronic Heart Failure

**DOI:** 10.1155/2021/7140044

**Published:** 2021-11-09

**Authors:** Qianyan Wu, Qingyuan Zhang, Yong Li, Letian Yu, Ying Zhang, Meiying Ao

**Affiliations:** ^1^Evidence-Based Medicine Research Center, College of Traditional Chinese Medicine, Jiangxi University of Chinese Medicine, Nanchang 330004, China; ^2^College of Traditional Chinese Medicine, Jiangxi University of Chinese Medicine, Nanchang 330004, China; ^3^The Affiliated Hospital of Jiangxi University of Chinese Medicine, Nanchang 330000, China

## Abstract

**Background:**

Chronic heart failure is the main critical illness and cause of death in the later stages of cardiovascular disease, and it is one of the two major challenges in the field of cardiovascular research. The clinical application of traditional Chinese medicine in the prevention and treatment of chronic heart failure has been relatively common in China, and the “Expert Consensus on the Diagnosis and Treatment of Chronic Heart Failure with Integrated Traditional Chinese and Western Medicine” has been published in China. Combining the literature in this field, the authors found that Zhigancao Decoction has been used in the treatment of chronic heart failure with more clinical research reports and higher frequency (this article refers to it as a high-frequency prescription for short). However, Zhigancao Decoction was not included in the recommended prescriptions in the “Expert Consensus on the Diagnosis and Treatment of Chronic Heart Failure with Integrated Traditional Chinese and Western Medicine,” and there was no relevant systematic review and meta-analysis. For this reason, this article has carried out two parts of work, including systematically organizing the literature in this research field and carrying out systematic review and meta-analysis. This can provide stronger evidence support for Zhigancao Decoction combined with conventional Western medicine in the treatment of chronic heart failure and provide a new option for the improvement and update of the “Expert Consensus on the Diagnosis and Treatment of Chronic Heart Failure with Integrated Traditional Chinese and Western Medicine.”

**Methods:**

This article used the bibliometric method to investigate the research articles on the treatment of chronic heart failure with integrated traditional Chinese and Western medicine and analyzed the high-frequency prescriptions which are used and reported frequently. In addition, we also used manual and computer-aided search methods, the search scope includes CNKI, WANFANG, VIP, SinoMed, Web of Science, PubMed, and Cochrane Library, and the search content is the clinical randomized control of Zhigancao Decoction combined with conventional Western medicine in the treatment of chronic heart failure trials (RCTs). The search period is from the establishment of the database to January 29, 2021. The literature was managed and screened by EndNote software; the quality of the included literature was evaluated according to the modified Jadad scale, and the risk bias was assessed using the Cochrane tool; the results of the included studies were analyzed using the Review Manager 5.3 software; the sources of heterogeneity between the studies were analyzed using Stata16.0 software for sensitivity analysis.

**Results:**

According to the bibliometric analysis, the maximum number of research reports is 553, which are arranged in descending order of 21 prescriptions, including Zhenwu Decoction, Zhigancao, and powder of five drugs containing poria. The second most frequently used prescription is Zhigancao Decoction combined with conventional Western medicine in the treatment of chronic heart failure, but its systematic review and meta-analysis still need further research. A total of 17 clinical randomized controlled trials of Zhigancao Decoction combined with conventional Western medicine in the treatment of chronic heart failure were included in the search, with a total of 1752 subjects. Meta-analysis results show that Zhigancao combined with conventional Western medicine is more effective than conventional Western medicine in the treatment of chronic heart failure. The advantages are the following 5 outcome indicators: total clinical effective rate, left ventricular ejection fraction, left ventricular end-diastolic diameter, B-type natriuretic peptide, and 6-minute walk test.

**Conclusions:**

There are many prescriptions combined with Western medicine to treat chronic heart failure, among which Zhigancao Decoction is the second most frequently used prescription. There are many original studies on Zhigancao Decoction combined with conventional Western medicine in the treatment of chronic heart failure. The quality of the evaluation research shows that the overall standard is scientific, and a few experimental designs are slightly irregular. Meta-analysis shows that Zhigancao Decoction combined with conventional Western medicine has better therapeutic effects and safety than conventional Western medicine. This shows the characteristics and advantages of integrated Chinese and Western medicine in the treatment of cardiovascular diseases and is worth recommending.

## 1. Introduction

Chronic heart failure is a complex clinical syndrome caused by ventricular systolic or diastolic disorders caused by abnormal structure or function of the heart. The main symptoms are dyspnea, fatigue, and fluid retention [1, 2]. Chronic heart failure is the main critical illness and cause of death in the later stages of cardiovascular disease. By 2017, it has affected the lives and health of nearly 2% of adults worldwide and is one of the two major challenges in the field of cardiovascular research [3, 4]. Western medicine focuses on pertinence in treating diseases. The current Western medicines for the treatment of chronic heart failure are mainly diuretics, neuroendocrine blockers, *β*-receptor antagonists, inotropic drugs, and vasodilators, etc., which are of great significance in improving myocardial function [5–8]. TCM treatment of diseases focuses on overall reconciliation, advocates syndrome differentiation and treatment, and emphasizes multitarget, multichannel, and multilink intervention, so as to achieve the effect of compatibility of all drugs and synergy [9]. Traditional Chinese medicine classifies chronic heart failure into the categories of “palpitations”, “asthma”, “edema”, “phlegm”, etc. It belongs to the syndrome of deficiency and excess, and the pathogenesis can be summarized as “deficiency”, “stasis”, and “water” [10, 11]. Chronic heart failure is a complex pathological process. The combined treatment of TCM and Western medicine not only improves local indicators but also regulates body functions as a whole. At present, clinical reports on the adjuvant treatment of chronic heart failure with TCM are increasing [12–15]. Based on the medication recommendations in the “Expert Consensus on the Diagnosis and Treatment of Chronic Heart Failure with Integrated Traditional Chinese and Western Medicine”, etc. [16, 17], this article first sorted out the clinical research and systematic review reports of its prescriptions for the treatment of chronic heart failure and selects its commonly used and unreviewed Zhigancao Decoction as the object of in-depth research.

Zhigancao Decoction, also known as Fumai Decoction, is a commonly used prescription for the treatment of chronic heart failure [18]. The original formula is made up of Zhigancao, *Rehmannia glutinosa*, *Ophiopogon japonicus*, cassia twig, ginseng, donkey-hide gelatin, hemp, ginger, and jujube [19]. The combination of total saponins of Ophiopogon japonicus, glycyrrhizic acid, and total ginsenosides can significantly reduce the autonomy and excitability of atrial muscles and prolong the refractory period of left atrial muscle function. Ginsenosides can increase the number of oxygen free radicals and inhibit renin-vascular tension. In addition, the cyclic adenosine monophosphate contained in jujube can slow down the ventricular rate [20]. The whole prescription of Zhigancao Decoction can shorten the action potential time course caused by low magnesium, reduce the excitability of the myocardium, significantly improve heart function and clinical symptoms, and effectively regulate the patient's cytokine level [21, 22]. At present, there are sufficient clinical randomized controlled trials of Zhigancao Decoction in the treatment of chronic heart failure, but there was no systematic review of relevant trials. The summary of clinical research on Zhigancao Decoction combined with conventional Western medicine in the treatment of chronic heart failure is still not clear. Therefore, this article will conduct a systematic evaluation and meta-analysis of Zhigancao Decoction combined with conventional Western medicine in the treatment of chronic heart failure, to scientifically evaluate the characteristic efficacy of combined Chinese and Western medicine. This can provide evidence support for the clinical application and promotion of combined Chinese and Western medicine for the treatment of the disease.

## 2. Methods

### 2.1. Research Registration

This research program has been registered in INPLASY, and the registration number is INPLASY202160098.

### 2.2. The Literature Retrieval Method of Clinical Research and Systematic Review of Prescriptions for Chronic Heart Failure

#### 2.2.1. Literature Search

Based on the prescriptions recommended by “Expert Consensus on the Diagnosis and Treatment of Chronic Heart Failure with Integrated Traditional Chinese and Western Medicine” and “Progress in the treatment of chronic heart failure with integrated traditional Chinese and Western medicine” [16, 17], we used the different recommended prescription and chronic heart failure as keywords or subject terms to edit formula for literature retrieval. In PubMed and CNKI databases, the retrieval formula is as follows, respectively, ((((((different recommended prescriptions [Title/Abstract]) AND (chronic heart failure[Title/Abstract])) OR (Chronic congestive heart failure[Title/Abstract])) OR (palpitations[Title/Abstract])) OR (edema[Title/Abstract])) OR (asthma[Title/Abstract])) OR (Phlegm[Title/Abstract]), SU = 各种被推荐的方剂^*∗*^(慢性心衰+慢性心力衰竭+慢性充血性心力衰竭+心悸+水肿+喘证+痰饮).

#### 2.2.2. Bibliometric Method

Through the literature count, we have counted the frequency of clinical research on various prescriptions for the treatment of chronic heart failure and the general situation of the research on the treatment of the disease with Chinese medicine to screen the prescriptions used frequently.

### 2.3. Zhigancao Combined with Conventional Western Medicine in the Treatment of Chronic Heart Failure Clinical Research and Treatment

#### 2.3.1. Search Strategy

From the computer and manual search of 7 Chinese and English databases, the search scopes are China Journal Full-text Database of China National Knowledge Infrastructure (CNKI), Wanfang Data Knowledge Service Platform (WANFANG), VIP Chinese Science and Technology Journal Full-text Database (VIP), Chinese Biomedical Literature Service System (SinoMed), Web of Science (WOS), PubMed, and Cochrane Library. The search content is all published randomized controlled trials (RCTs) of Zhigancao combined with conventional Western medicine in the treatment of chronic heart failure. The search time span is from the establishment of the database to January 29, 2021. The search strategy is flexibly adjusted according to the characteristics of the major databases. The main search terms include “Zhigancao”, “ZhiGancao Soup”, “Roast Radix *Glycyrrhiza* Decoction”, “Fumai Decoction”, “Fumai Soup”, “Chronic Congestive Heart Failure”, “CHF”, and “Chronic Heart Failure”.

Take the PubMed Database search as an example. Other databases adopt similar search strategies:

1. #1 “Zhigancao Decoction” + “Zhigancao soup” + “Added Zhigancao” + “Fumai Decoction” + “Fumai Decoction plus and minus” + “Fumai Decoction”
2. #2 “Chronic Heart Failure” + “Chronic Congestive Heart Failure” + “Edema” + “Asthma” + “Phlegm” + “Palpitation”
3. #3 “#1”^*∗*^”#2″”

#### 2.3.2. Inclusion Criteria

1. Document type: clinical randomized controlled trials (RCTs) of Zhigancao Decoction combined with conventional Western medicine methods for the treatment of chronic heart failure published in domestic and foreign journals; the time, region, and area of publication of the literature; not restricted by blinding or allocation concealment methods; the languages of the literature are Chinese and English.
2. Subjects of the study: in the original study, the subjects were clearly diagnosed with chronic heart failure; the specific diagnostic criteria are as follows: syndrome types are not limited; race, nationality, and gender are not limited; and the age is required to be greater than 18 years old.
3. Intervention measures: the control group was treated with conventional Western medicine methods, including oxygen inhalation, diuretics, angiotensin receptor antagonists, and *β*-receptor blockers; the treatment group was treated with traditional Chinese medicine on the basis of the control group's medications symptomatic treatment with Zhigancao Decoction.
4. Outcome indicators: total clinical efficacy, left ventricular ejection fraction (LVEF), left ventricular end-diastolic diameter (LVEDd), B-type natriuretic peptide (BNP), and 6-minute walk test (6MWT). The outcome indicators include at least one of the five outcome indicators.

#### 2.3.3. Exclusion Criteria

1. Patients with other serious diseases
2. Nonclinical randomized controlled trials such as review, cell animal and other nonclinical trials, clinical case reports, experience summaries of famous doctors, and theoretical analysis
3. The trial design plan is not rigorous, and the statistical method is inappropriate
4. Incomplete original data or obvious errors in the data
5. Repeated reports and suspected plagiarism
6. Unable to obtain full-text documents
7. Outcome indicators and intervention measures that do not meet the requirements

### 2.4. Meta-Analysis Methods of Zhigancao Decoction Combined with Conventional Western Medicine in the Treatment of Chronic Heart Failure

Meta-analysis methods are drawn from the literature [23–25], including screening and data extraction, literature quality assessment, and statistical methods.

## 3. Results

### 3.1. The Results of Systematic Literature Reviews on the Clinical Studies of Prescriptions in the Treatment of Chronic Heart Failure

**Figure 1:**
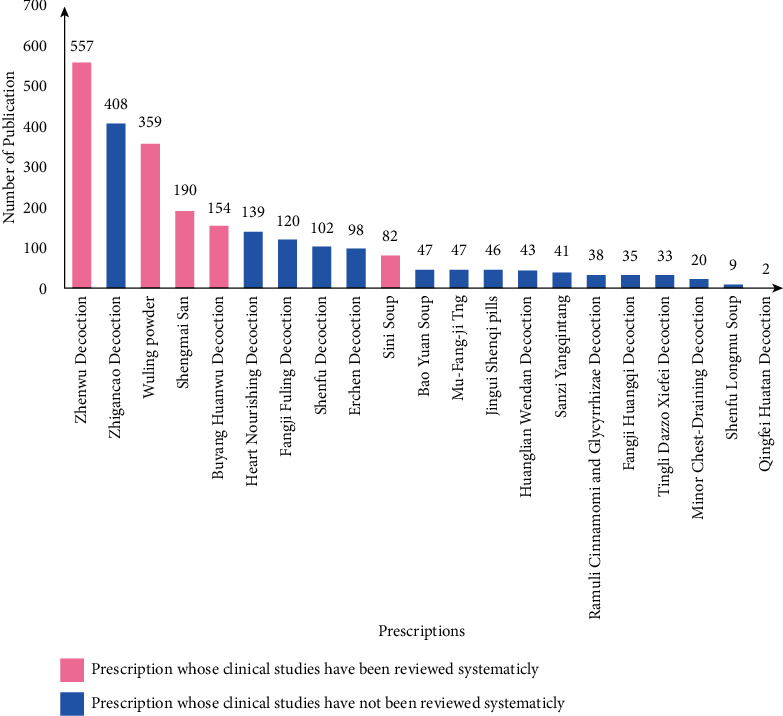
Metrological histogram of research reports on the treatment of chronic heart failure with Chinese medicine prescriptions.

Based on the recommended prescriptions in the “Expert Consensus on the Diagnosis and Treatment of Chronic Heart Failure with Integrated Traditional Chinese and Western Medicine” and “Progress in the Treatment of chronic heart failure with integrated traditional Chinese and Western medicine” [16, 17], we chose the Chinese and English databases CNKI and PubMed as the categories for search, used “the recommended prescriptions” and “chronic heart failure” as keywords or subject terms for search, and conducted a bibliometric analysis for these retrieved publications about the recommended prescriptions. The result of retrieval is shown in Table 1. As shown in Table 1, there are up to 557, 408, 359, and 190 research reports, respectively, related to Zhenwu Decoction, Zhigancao Decoction, Wuling Powder, and Shengmai Powder. By 2020, there have been several systematic reviews and meta-analyses in which Zhenwu Decoction and Wuling Powder were used as the main intervention measures, which proved the clinical efficacy of these prescriptions in the treatment of chronic heart failure [26, 27]. To show the research reports and systematic reviews of different recommended prescriptions more intuitively, we drew a bar chart in which the red histogram represents prescriptions whose clinical studies have been reviewed systematically, and the blue histogram represents prescriptions whose clinical studies have not been reviewed systematically (Figure 1).

It is worth noting that Zhigancao Decoction's number of literature reports on the treatment of chronic heart failure ranks second and is the first among these prescriptions whose clinical studies have not been reviewed systematically (Figure 1). In addition, the systematic reviews and meta-analysis of Zhigancao Decoction treating chronic heart failure had not been retrieved yet while the categories of the search were expanded to seven commonly used Chinese and English databases. Therefore, the systematic review and meta-analysis of Zhigancao Decoction treating chronic heart failure were carried out for the first time in this research based on its clinical studies.

### 3.2. Retrieval of Clinical Research on Zhigancao Decoction Combined with Conventional Western Medicine in the Treatment of Chronic Heart Failure and the Quality of the Included Literature

Based on the evidence-based method, we collected all clinical randomized controlled studies of “Zhigancao Decoction combined with conventional Western medicine in the treatment of chronic heart failure”. The retrieval and inclusion process are as follows.

#### 3.2.1. Retrieval Flowchart

**Figure 2:**
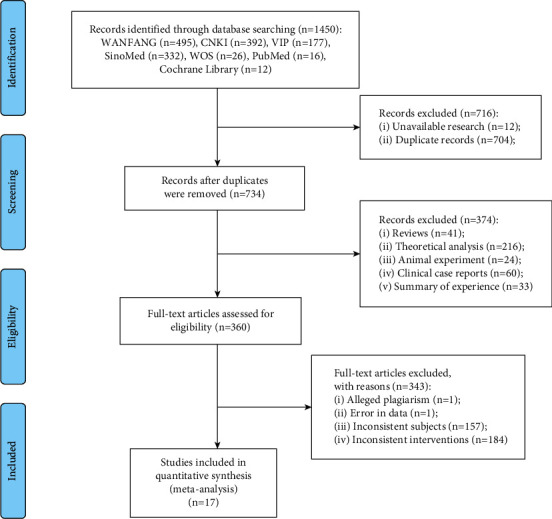
Flow chart of research literature selection.

By searching 7 commonly used Chinese and English databases, we obtained 1,450 articles, including 392 articles in CNKI, 495 articles in WANGFAN, 177 articles in VIP, 332 articles in SinoMed, 26 articles in Web of Science, 16 articles in PubMed, and 12 articles in Cochrane Library. According to the criteria and procedures for inclusion and exclusion, a total of 1433 articles were removed, including 12 articles without full texts, 704 duplicate articles, 41 reviews, 33 experience summaries of famous doctors, 24 cell animal nonclinical trials, 60 clinical case reports, 1 study involving plagiarism, 1 study with obvious data errors, 216 theoretical analyses, 184 studies in which intervention measures did not meet the requirements, and 157 studies in which the research objects did not meet the requirements. Finally, 17 randomized controlled trials that met the standards were obtained [28–44]. The literature screening process is shown in Figure 2.

#### 3.2.2. Characteristics of Included Studies

A total of 17 clinical randomized controlled trials were included in this study [28–44]. A total of 1752 patients with chronic heart failure were included, 890 in the treatment group and 862 in the control group. The basic characteristics of the included research literature are shown in Table 2.

#### 3.2.3. Results of Literature Quality Evaluation Included

**Figure 3:**
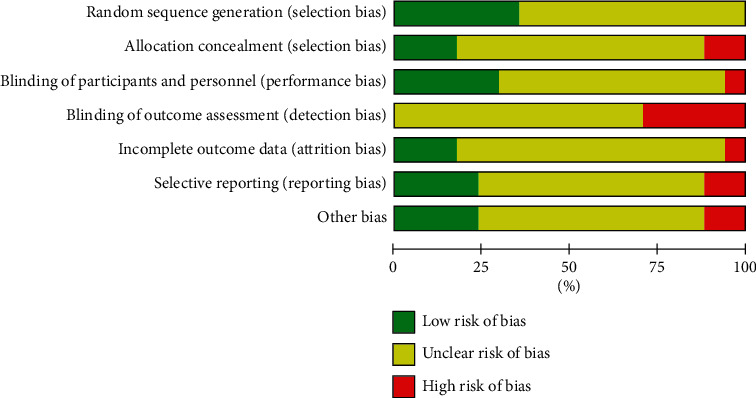
The distribution of the risk of bias in the included research literature.

**Figure 4:**
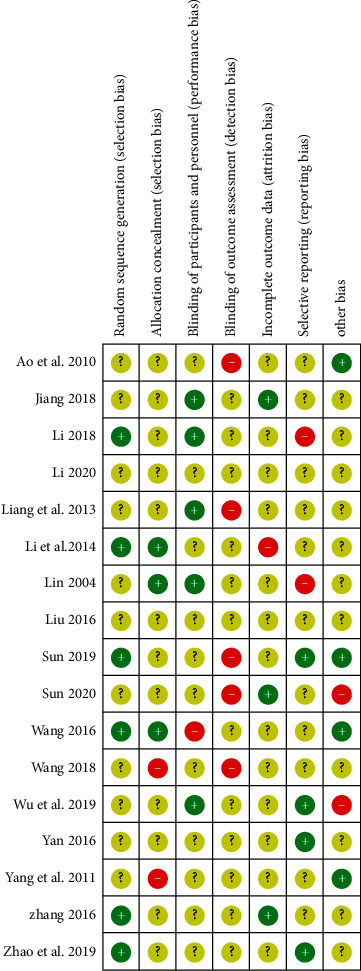
The bias of the included research literature.

The 17 included literature studies were grouped by the random method, of which 4 studies [29, 39, 41, 44] used the random number table method, 2 studies [32, 33] used the simple random method, 1 study [38] used the odd and even number random method, 1 study [43] used the two-color ball random method, and 1 study [33] reported on the allocation concealment method and whether there was an exit event. The scores of the included literature studies did not exceed 3, and the methodological quality scores of the included literature studies are shown in Table 3. In addition, according to the risk bias evaluation tool provided by the Cochrane Collaboration, the included literature is evaluated for bias risk, as shown in Figures 3 and 4.

### 3.3. Meta-Analysis of Measured Outcomes

#### 3.3.1. Clinical Effective Rate

**Figure 5:**
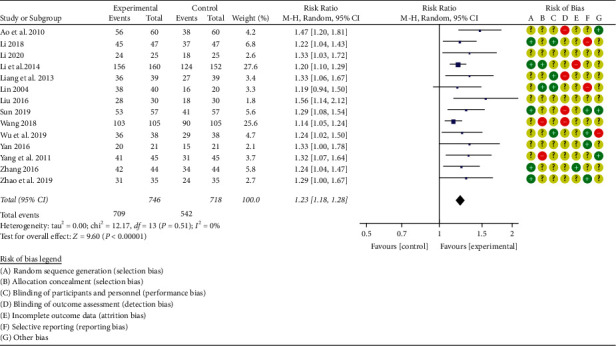
Forest plot of the total clinical effective rate.

A total of 14 studies [28–32, 34, 36–39, 41–43] reported the total clinical effective rate (see Figure 5). A total of 1464 patients with chronic heart failure were included, with 746 patients in the treatment group and 718 patients in the control group. The random-effects model was used to conduct a meta-analysis, and the study was tested for heterogeneity (*P* < 0.00001, I^2^ = 0%).

#### 3.3.2. Left Ventricular Ejection Fraction (LVEF) Level

**Figure 6:**
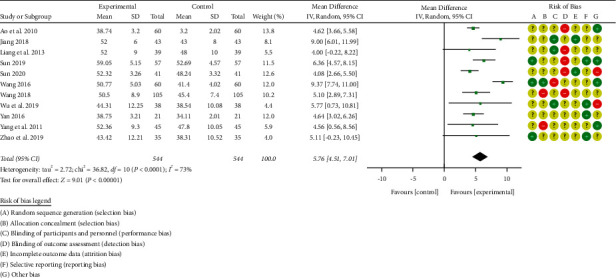
Forest plot of left ventricular ejection fraction (LVEF).

There are 11 studies [28, 31, 33–37, 40–41, 43–44] that reported the levels of left ventricular ejection fraction (LVEF) before and after treatment (see Figure 6). A total of 1088 patients with chronic heart failure were included, 544 cases in the treatment group and 544 cases in the control group. There was significant heterogeneity among the studies (*P* < 0.00001, I^2^ = 73%), so the random-effects model was used for integrated analysis.

#### 3.3.3. Left Ventricular End-Diastolic Dimension (LVEDd) Level

**Figure 7:**
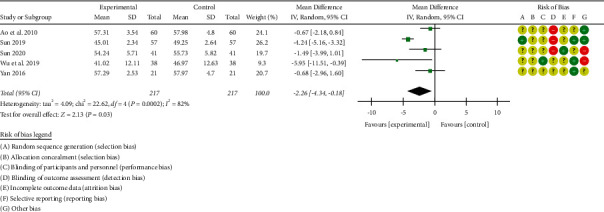
Forest plot of left ventricular end-diastolic diameter (LVEDd).

A total of 5 studies [28, 31, 40–41, 44] reported the improvement of the left ventricular end-diastolic diameter (LVEDd) after treatment. There were 434 patients with chronic heart failure, including 217 in the treatment group and 217 in the control group. There was significant heterogeneity among the studies [*P* < 0.00001, I^2^ = 82%], so the random-effects model was used for integrated analysis. Meta-analysis results were as follows. The left ventricular end-diastolic diameter (LVEDd) forest plot shows that the diamond is located to the left of the invalid vertical line (see Figure 7), combined with the effect size [WMD = −2.26, 95%CI (−4.34, −0.18), *P* < 0.00001]; the difference between the two groups was statistically significant. This shows that roasted Zhigancao Decoction combined with conventional Western medicine treatment improves the level of left ventricular end-diastolic diameter (LVEDd), which is better than using conventional Western medicine alone.

#### 3.3.4. B-Type Natriuretic Peptide (BNP) Level

**Figure 8:**
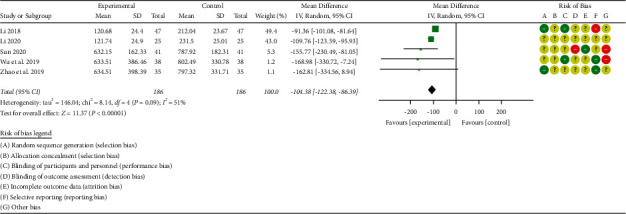
Forest plot of B-type natriuretic peptide (BNP).

A total of 5 studies [38–40, 43–44] reported levels of B-type natriuretic peptide (BNP) before and after treatment, with a total of 372 subjects, including 186 cases in the treatment group and 186 cases in the control group. There was significant heterogeneity among the studies [*P* < 0.00001, I^2^ = 51%], so the random-effects model was used for integrated analysis. Meta-analysis results were as follows: B-type natriuretic peptide (BNP) forest plot shows that the diamond is located on the left side of the invalid vertical line (see Figure 8), combined with the analysis data [WMD = −104.38, 95%CI (−122.38, −86.39), *P* < 0.00001]; the difference between the groups is statistically significant. This shows that the traditional Chinese medicine Zhigancao Decoction combined with the conventional Western medicine treatment of chronic heart failure improves the level of B-type natriuretic peptide (BNP), which is better than the control group.

#### 3.3.5. 6-Minute Walk Test (6MWT) Level

**Figure 9:**
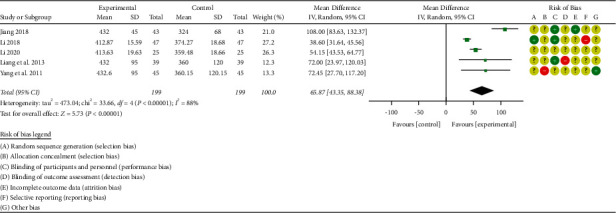
Forest plot of the 6-minute walk test (6MWT).

A total of 5 studies with the outcome index of the 6-minute walk test (6MWT) level were included [33–36, 38–39]. There were 398 patients with chronic heart failure, including 199 in the treatment group and 199 in the control group. There was significant heterogeneity among the studies [*P* < 0.00001, I^2^ = 88%], so the random-effects model was used for integrated analysis. Meta-analysis results were as follows: 6-minute walk test (6MWT) forest plot shows that the diamond is located on the right side of the invalid vertical line (see Figure 9), combined with the effect size [WMD = 65.87, 95%CI (43.35, 88.38), *P* < 0.00001]. The differences between groups are statistically significant. This shows that Zhigancao Decoction combined with conventional Western medicine can improve the 6-minute walk test (6MWT) level, which is better than using conventional Western medicine alone.

#### 3.3.6. Source Analysis of Heterogeneity

**Figure 10:**
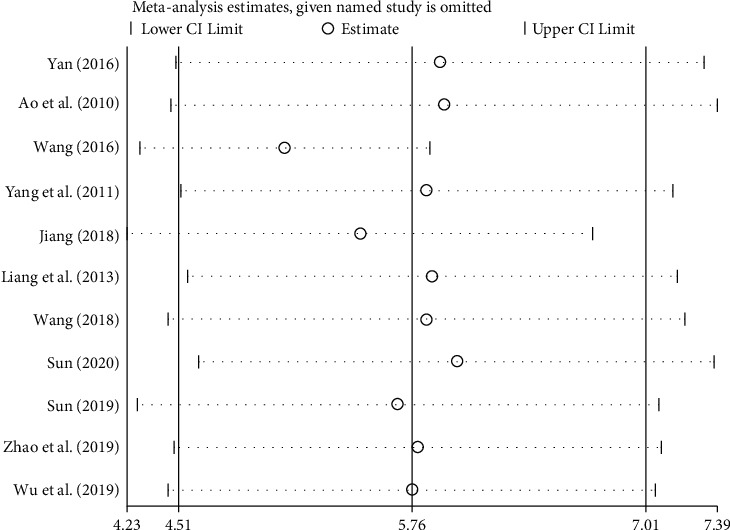
Sensitivity analysis graph of left ventricular ejection fraction (LVEF).

**Figure 11:**
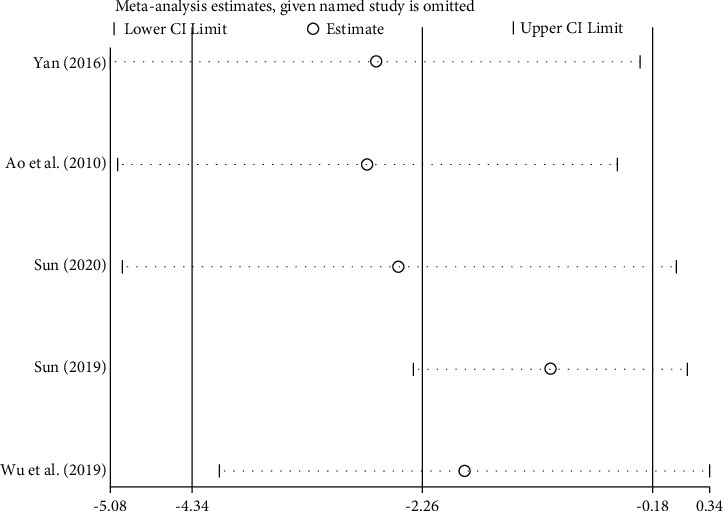
Sensitivity analysis chart of left ventricular end-diastolic diameter (LVEDd).

**Figure 12:**
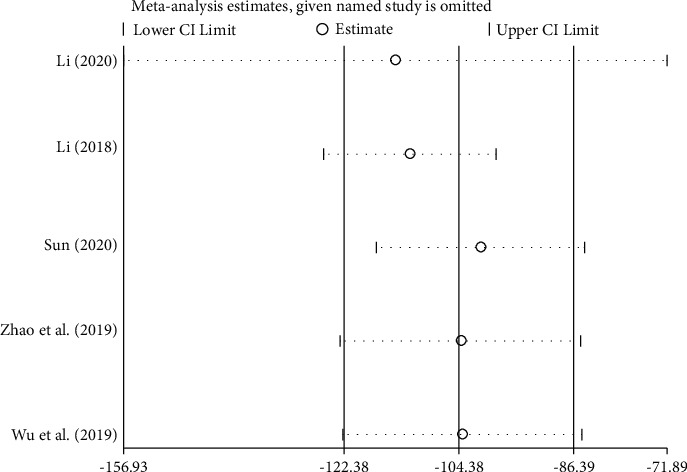
Sensitivity analysis diagram of B-type natriuretic peptide (BNP).

**Figure 13:**
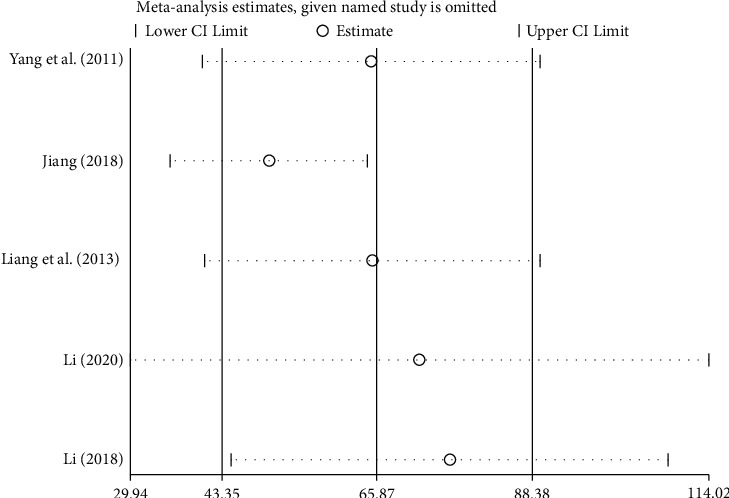
Sensitivity analysis diagram of the 6-minute walk test (6MWT).

Sensitivity analysis of 4 outcome indicators includes left ventricular ejection fraction (LVEF), left ventricular end-diastolic diameter (LVEDd), B-type natriuretic peptide (BNP), and 6-minute walk test (6MWT), as shown in Figures 10–13. The sensitivity analysis graph of left ventricular ejection fraction (LVEF) shows that Wang's [33] analysis line is significantly biased, so Wang [33] was the main source of heterogeneity of the outcome index. The left ventricular end-diastolic diameter (LVEDd)) sensitivity analysis diagram shows that Sun Tiancai [41] was the main source of heterogeneity of the index; B-type natriuretic peptide (BNP) sensitivity analysis diagram shows that Li [38] was the possible main heterogeneity source: the sensitivity analysis chart of the 6-minute walk test (6MWT) shows that Jiang [35] was the main source of possible heterogeneity. These sources of heterogeneity were related to the period of the disease between studies, the dosage of Zhigancao Decoction, and the number of samples in the experiment.

#### 3.3.7. Publication Bias

**Figure 14:**
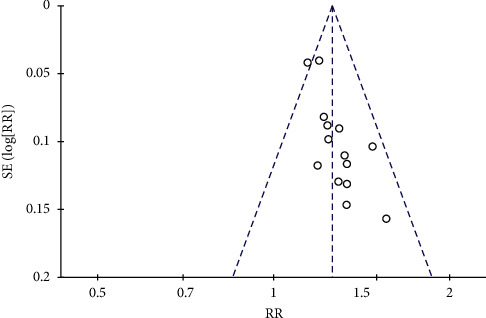
Funnel diagram of the total clinical effectiveness of publication.

**Figure 15:**
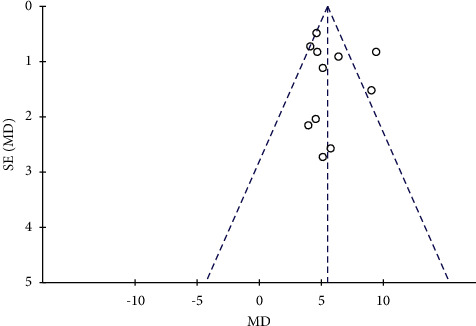
Funnel diagram of the bias of the publication about the left ventricular ejection fraction (LVEF).

The total clinical effective rate and the left ventricular ejection fraction (LVEF) level were analyzed in an inverted funnel chart to observe the distribution of the inverted funnel chart. The total clinical effective rate inverted funnel chart equivalent line is scattered on both sides, there is a slight deviation, and the symmetry is small, as shown in Figure 14. This indicates that there is publication bias. This is because of the low quality of the included clinical randomized controlled trials and the selective reporting of trial data. The inverted funnel plot of the left ventricular ejection fraction (LVEF) shows basic symmetry, and the scattered points of the research data are evenly distributed on both sides of the straight line, as shown in Figure 15. This shows that there is no significant publication bias for included studies.

#### 3.3.8. Adverse Reactions and Safety

Two studies reported the specific occurrence of clinical adverse reactions (see Table 4). Among them, Zhao et al. [43] showed that the adverse reaction symptoms were significantly relieved after symptomatic treatment. The results of routine blood routine, urine routine, liver and kidney function, and electrolytes before and after treatment showed no abnormalities. Wang [33] and Li [39] showed that there were no serious adverse reactions in the treatment group and the control group. Therefore, Zhigancao Decoction combined with conventional Western medicine treatment of chronic heart failure has good and reliable safety.

## 4. Conclusions and Discussion

### 4.1. Zhigancao Decoction Is a Common Prescription in the Treatment of Chronic Heart Failure, Which Can Improve the Hematopoietic Function of the Heart and Protect the Myocardium

Firstly, the studies of Zhigancao Decoction treating chronic heart failure are sufficient, whose number of being publicated is up to 408 in CNKI and PubMed databases, and Zhigancao Decoction is the second high-frequency prescription among these recommended prescriptions in the treatment of chronic heart failure (Table 1 and Figure 1). Secondly, Zhigancao Decoction is derived from “Treatise on Febrile Diseases” written by Zhang Zhongjing in Han Dynasty and can activate qi, supplement blood, and return pulse [45]. In recent years, many basic experiment studies proved that Zhigancao Decoction did improve the hematopoietic function of the heart and protect the myocardium. As reported, Zhigancao Decoction can inhibit excessive autophagy and reduce self-injury of myocardial cells by means of lowering the abnormally high level of myocardial enzymes of CK, LDH, and AST, regulating the phosphorylation level of Akt and mTOR positively, and increasing the transcription level of PI3K, Akt, and mTOR genes remarkably [46]. Zhigancao Decoction can also help to generate blood and improve the hematopoietic function of the heart by stimulating the secretion of TPO by umbilical vein endothelial cells, stimulating the differentiation of hematopoietic stem cells into megakaryocyte progenitor cells, and promoting the proliferation and differentiation of megakaryocyte progenitor cells and the maturation of megakaryocytes [47, 48].

### 4.2. The Clinical Research Quality of Zhigancao Decoction Combined with Conventional Western Medicine in the Treatment of Chronic Heart Failure Is Acceptable

A total of 7 commonly used Chinese and English databases were searched, including Chinese and English tests. The amount of literature and data available for research was sufficient. The literature retrieval process uses detailed and strict retrieval strategies and inclusion criteria to minimize publication bias. The included 17 clinical randomized controlled trials are all Chinese studies, and the study sites are all in China. Research on countries and regions that also widely use Chinese medicine to treat diseases, such as Japan and South Korea, is still unclear. The methodology of the research is a holistic science. A small number of experimental designs are slightly irregular, which may affect the overall research situation. In addition, due to the differences in conventional Western medicine treatment methods adopted by each treatment group, the compatibility and administration of Zhigancao Decoction, treatment, age, gender, condition of the subjects, and pathogenic factors, these factors may affect the credibility of the meta-analysis results.

### 4.3. Zhigancao Decoction Combined with Conventional Western Medicine  Has Better Clinical Efficacy and Safety in the Treatment of Chronic Heart Failure

The included 17 clinical randomized controlled trials were all Western medicine conventional method combined with Zhigancao Decoction for the treatment of chronic heart failure. The study includes multiple outcome indicators, and a comprehensive clinical treatment effect evaluation can be obtained through a comprehensive and systematic analysis. The combined test of outcome indicators showed that the combined treatment of Chinese and Western medicine can significantly improve the clinical effectiveness of chronic heart failure, the left ventricular ejection fraction (LVEF) level, the left ventricular end-diastolic diameter (LVEDd) level, B-type natriuretic peptide (BNP) level, and 6-minute walk test (6MWT) level. The clinical efficacy and safety of combined treatment of traditional Chinese and Western medicine are better than those of conventional methods of Western medicine alone.

The medicated serum of Zhigancao Decoction can inhibit ICa-L and has a good regulatory effect on calcium channels in internal organs such as the myocardium and thoracic aorta. Its mechanism of action is the same as that of Western medicine calcium channel blockers [49]. Therefore, Zhigancao Decoction combined with conventional Western medicine treatment of chronic heart failure can jointly improve the relevant clinical symptoms of patients, optimize myocardial energy metabolism, improve heart function, and improve ventricular pumping or filling function [50, 51].

In conclusion, the clinical application of combined Chinese and Western medicine in the treatment of chronic heart failure has been widely used in China, and there are many related prescriptions. In addition, there are many clinical reports about Zhigancao Decoction combined with conventional Western medicine in the treatment of chronic heart failure. Meta-analysis results show that Zhigancao Decoction combined with conventional Western medicine has better therapeutic effects and safety than only using conventional Western medicine. This shows the characteristics and advantages of the combination of Chinese and Western medicine in treating cardiovascular diseases and is worth recommending.

## Figures and Tables

**Table 1:** Systematic evaluation and meta-analysis of traditional Chinese medicine in the treatment of chronic heart failure.

No.	Prescription	Number of articles in CNKI and PubMed	Is there a systematic review and meta-analysis report	Quantity	Most recently reported year
1	Zhenwu Decoction	557	Yes	7	2020
2	Zhigancao Decoction	408	No	0	—
3	Wuling Powder	359	Yes	4	2020
4	Shengmai San	190	Yes	2	2019
5	Buyang Huanwu Decoction	154	Yes	1	2020
6	Heart Nourishing Decoction	139	No	0	—
7	Fangji fuling Decoction	120	No	0	—
8	Shenfu Decoction	102	No	0	—
9	Erchen Decoction	98	No	0	—
10	Sini soup	82	Yes	6	2020
11	Bao Yuan soup	47	No	0	—
12	Mu-Fang-Ji Tang	47	No	0	—
13	Jinkui Shenqi Pills	46	No	0	—
14	Huanglian Wendan Decoction	43	No	0	—
15	Sanzi Yangqintang	41	No	0	—
16	Ramuli Cinnamomi and Glycyrrhizae Decoction	38	No	0	—
17	Fangji Huangqi Decoction	35	No	0	—
18	Tingli Dazao Xiefei Decoction	33	No	0	—
19	Minor Chest-Draining Decoction	20	No	0	—
20	Shenfu Longmu soup	9	No	0	—
21	Qingfei Huatan Decoction	2	No	0	—

**Table 2:** Basic characteristics of the included research literature.

Study or subgroup	Sample (T/C)	Age		Prescription		Course of treatment	Outcome indicators
		T	C	C	T		
Yan 2016 [28]	21/21	72.5 ± 5.3	70.5 ± 5.4	Digoxin tablets	Add Zhigancao soup	24 w	①②③
Li et al. 2014 [29]	160/152	52–81, mean 60.2	51–82, mean 60.6	Amiodarone hydrochloride tablets	Add Zhigancao soup	4 w	①
Liu 2016 [30]	30/30	75 ± 5	72 ± 3	Digoxin tablets	Add Zhigancao soup	6 w	①
Ao et al. 2010 [31]	60/60	72 ± 10	72 ± 10	Digoxin tablets	Add Zhigancao soup	4 w	①②③
Zhang 2016 [32]	44/44	70.69 ± 3.25	70.69 ± 3.23	Digoxin tablets	Add Zhigancao soup	4 w	①
Wang 2016 [33]	60/60	64.2 ± 12.0	66.9 ± 11.9	Hydrochlorothiazide, enalapril, digoxin tablets	Add Zhigancao soup	45 d	③
Yang et al. 2011 [34]	45/45	57–84, mean 69	57–84, mean 69	Oxygen inhalation, digoxin, furosemide, dihydrocurethiazide, spironolactone, enalapril, isosorbide, betaloc	Add Zhigancao soup	12 w	①③⑤
Jiang 2018 [35]	43/43	58.5 ± 1.5	61.5 ± 2.5	Betaloc, isosorbide, enalapril, spironolactone, furosemide, digoxin, oxygen inhalation	Add Zhigancao soup	4 w	③⑤
Liang et al. 2013 [36]	39/39	55–82, mean 68	55–82, mean 68	Betaloc, isolator, enalapril, spironolactone, dihydrocurethiazide, furosemide, digoxin, oxygen inhalation	Add Zhigancao soup	12 w	①③⑤
Wang 2018 [37]	105/105	61.4 ± 2.9	62.8 ± 3.1	Metoprolol	Add Zhigancao soup	12 w	①③
Li 2020 [38]	25/25	50–80	50–79	Metoprolol tartrate tablets, sacubitril, and valsartan sodium tablets	Add Zhigancao soup	Unmentioned	①④⑤
Li 2018 [39]	47/47	60.31 ± 7.62	60.44 ± 7.71	Betaloc, digosine tablets, sacubitril and Valsartan sodium tablets, spironolactone tablets	Add Zhigancao soup	2 w	①④⑤
Sun 2020 [40]	41/41	65.82 ± 14.92	64.36 ± 15.92	Furosemide tablets, perindopril tert-butylamine tablets, metoprolol tartrate tablets, digoxin tablets, aspirin enteric-coated tablets, simvastatin tablets	Add Zhigancao soup	Unmentioned	②③④
Sun 2019 [41]	57/57	58.64 ± 6.21	57.68 ± 6.13	Vasartan	Add Zhigancao soup	12 w	①②③
Lin 2004 [42]	40/20	61–76	60–78	Routine treatment for oxygen inhalation, heart strengthening, diuresis, and circulation improvement	Add Zhigancao soup	Unmentioned	①
Zhao et al. 2019 [43]	35/35	54.31 ± 3.82	54.25 ± 3.56	Routine treatments such as diuretics, angiotensin receptor antagonists, angiotensin converting enzyme inhibitors, *β*-receptor blockers, and aldosterone receptor antagonists	Add Zhigancao soup	4 w	①③④
Wu et al. 2019 [44]	38/38	55.27 ± 2.63	55.38 ± 2.54	Digoxin, metoprolol tartrate tablets, hakubatril, and valsartan sodium tablets	Add Zhigancao soup	Unmentioned	①②③④

*Note.* T/C: test group/control group; ①: total clinical effective rate; ②: left ventricular end-diastolic diameter; ③: left ventricular ejection fraction; ④: B-type natriuretic peptide; ⑤: 6 minutes of walking distance.

**Table 3:** Quality evaluation of included studies.

Study or subgroup	Random method	Randomized hiding	Blinding	Lost to follow-up/exit	Jadad score
Yan 2016 [28]	Unknown	Not mentioned	Not mentioned	Not mentioned	1
Li et al. 2014 [29]	Random number table	Not mentioned	Not mentioned	Not mentioned	2
Liu 2016 [30]	Unknown	Not mentioned	Not mentioned	Not mentioned	1
Ao et al. 2010 [31]	Unknown	Not mentioned	Not mentioned	Not mentioned	1
Zhang 2016 [32]	Simple random method	Not mentioned	Not mentioned	Not mentioned	2
Wang 2016 [33]	Simple random method	Disease sign group	Not mentioned	No dropout cases	3
Yang et al. 2011 [34]	Unknown	Not mentioned	Not mentioned	Not mentioned	1
Jiang 2018 [35]	Unknown	Not mentioned	Not mentioned	Not mentioned	1
Liang et al. 2013 [36]	Unknown	Not mentioned	Not mentioned	Not mentioned	1
Wang 2018 [37]	Unknown	Not mentioned	Not mentioned	Not mentioned	1
Li 2020 [38]	Odd and even number random method	Not mentioned	Not mentioned	Not mentioned	2
Li 2018 [39]	Random number table	Not mentioned	Not mentioned	Not mentioned	2
Sun 2020 [40]	Unknown	Not mentioned	Not mentioned	Not mentioned	1
Sun 2019 [41]	Random number table	Not mentioned	Not mentioned	Not mentioned	2
Lin 2004 [42]	Unknown	Not mentioned	Not mentioned	Not mentioned	1
Zhao et al. 2019 [43]	Two-color ball random method	Not mentioned	Not mentioned	Not mentioned	2
Wu et al. 2019 [44]	Random number table	Not mentioned	Not mentioned	Not mentioned	2

**Table 4:** The occurrence of adverse reactions.

Study or subgroup	Occurrence of adverse reactions		Adverse reaction rate	
	T	C	T	C
Sun 2020 [40]	3 cases of nausea and vomiting, 1 case of diarrhea, 1 case of headache, 1 case of rash	2 cases of nausea and vomiting, 1 case of diarrhea, 1 case of headache	14.63%	9.76%
Zhao et al. 2019 [43]	2 cases of nausea and vomiting, 1 case of diarrhea	2 cases of nausea and vomiting, 1 case of abdominal pain, 1 case of dizziness	8.57%	11.43%

## Data Availability

The data used to support the findings of this study are available from the corresponding author upon request.

## Supplementary Materials

Supplementary Materials Supplementary File 1: INPLASY registration checklist.
