# Supplementary material for: A Systematic Review and Meta-Analysis of High-Frequency Prescription of Zhigancao Decoction Combined with Conventional Western Medicine in the Treatment of Chronic Heart Failure
Source: Evid Based Complement Alternat Med. 2021 Nov 9;2021:7140044. doi: 10.1155/2021/7140044 (PMC8595005; doi:10.1155/2021/7140044)
Supplement: Supplementary Materials — Supplementary File 1: INPLASY registration checklist. [file 7140044.f1.pdf]

INPLASY - International Platform of Registered Systematic Review and Meta-analysis Protocols

INVOICE PUBLICATION FEES - $20.00

PAID

Billing Information

吴千言
1165382022@qq.com
Center for Evidence-Based and Translational Medicine, School of Traditional Chinese Medicine, Jiangxi University of Chinese Medicine
China

Payment Details

Payment Method: WeChat Pay
Status: Authorized

Invoice Number: **202160098**

Protocol: A systematic review and meta-analysis of high-frequency prescription of Zhigancao decoction combined with conventional western medicine in the treatment of chronic heart failure.

Date: 26 June 2021
E-mail: editorialmanager@inplasy.com
Address - INPLASY, Inc. 651 N Broad St, Suite 206, Middletown, DE 19709 USA.

INPLASY
